# Supplementary material for: Identification of photoperiod-induced specific miRNAs in the adrenal glands of Sunite sheep (Ovis aries)
Source: Front Vet Sci. 2022 Jul 22;9:888207. doi: 10.3389/fvets.2022.888207 (PMC9354845; doi:10.3389/fvets.2022.888207)
Supplement: Supplementary file 1 [file Data_Sheet_1.ZIP › Supplementary file/miRDeep2 file.zip!/miRDeep2 file/Novel_Structure/SPLP42A2/Novel_329.pdf]

Provisional ID	:	NC_019479.2_37940
Score total	:	1.8
Score for star read(s)	:	-1.3
Score for read counts	:	0
Score for mfe	:	1.5
Score for randfold	:	1.6
Score for cons. seed	:	
Total read count	:	254
Mature read count	:	254
Loop read count	:	0
Star read count	:	0

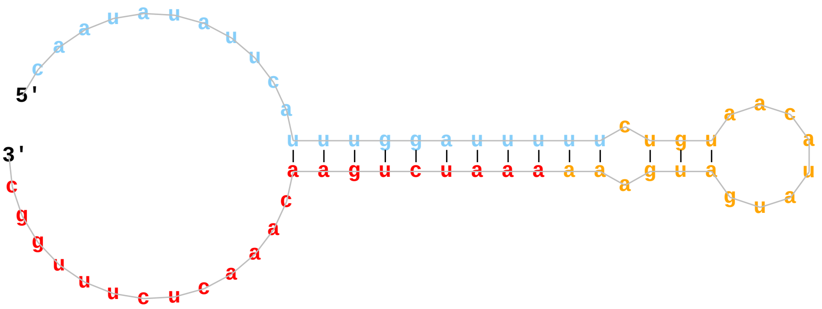

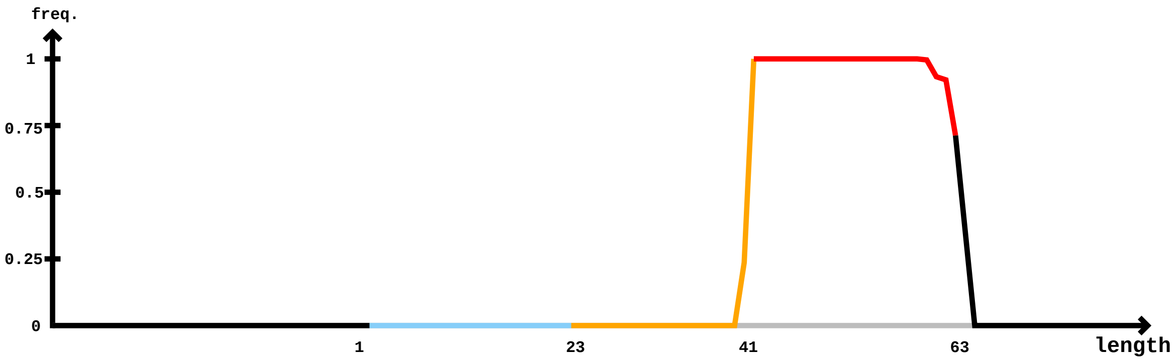

Star Mature

```
5'- ugcaagaucuggaacaagguauuggguuggccaauauauucauuuggauuuuucuguaacauaugaugaaaaaaucugaacaaacucuuuggccaacccaauacuaugagga   -3'   exp
    ..............((.(((((((((((((((((........(((((((((((.(((........))).)))))))))))........))))))))))))))))).))....   reads   mm   sample
    ......................................................................aaaaucugaacaaacuUuu.......................   1       1    seq
    ......................................................................aaaaucugaacaaacuUuuu......................   15      1    seq
    ......................................................................aaaaucugaacaaacucuuugg....................   1       0    seq
    ......................................................................aaaaucugaacaaacuUuuugg....................   39      1    seq
    ......................................................................aaaaucugaacaaacuUuuuggc...................   4       1    seq
    .......................................................................aaaucugaacaaacuUuuu......................   1       1    seq
    .......................................................................aaaucugaacaaacuUuuug.....................   3       1    seq
    .......................................................................aaaucugaacaaacuUuuugg....................   13      1    seq
    .......................................................................aaaucugaacaaacuUuuuggc...................   177     1    seq
```
